# Radiotherapy for hormone-sensitive prostate cancer with synchronous low burden of distant metastases

**DOI:** 10.1007/s00066-022-01961-y

**Published:** 2022-06-15

**Authors:** Arndt-Christian Müller, Daniel M. Aebersold, Clemens Albrecht, Dirk Böhmer, Michael Flentje, Ute Ganswindt, Pirus Ghadjar, Nina-Sophie Schmidt-Hegemann, Stefan Höcht, Tobias Hölscher, Peter Niehoff, Michael Pinkawa, Felix Sedlmayer, Frank Wolf, Constantinos Zamboglou, Daniel Zips, Thomas Wiegel, Prostate Cancer Expert Panel of the German Society of Radiation Oncology (DEGRO) and the Working Party Radiation Oncology of the German Cancer Society (DKG-ARO)

**Affiliations:** 1 Department of Radiation Oncology, Eberhard Karls University, Tübingen, Germany; 2 Department of Radiation Oncology, RKH-Kliniken, Posilipostr. 4, 71640 Ludwigsburg, Germany; 3 Department of Radiation Oncology Inselspital, University of Bern, Bern, Switzerland; 4 Klinik für Strahlentherapie, Klinikum Nürnberg Nord, Nürnberg, Germany; 5 Department of Radiation Oncology, Charité Universitätsmedizin Berlin, Berlin, Germany; 6 Department of Radiation Oncology, Universitätsklinikum Würzburg, Würzburg, Germany; 7 Department of Radiation Oncology, University Hospital Innsbruck, Innsbruck, Austria; 8 Department of Radiation Oncology, LMU Munich, Munich, Germany; 9 Xcare Praxis für Strahlentherapie Saarlouis, Xcare Gruppe, Saarlouis, Germany; 10 Department of Radiotherapy and Radiation Oncology, University Hospital Carl Gustav Carus, Technische Universität Dresden, Dresden, Germany; 11 Sana Klinikum Offenbach, Offenbach, Germany; 12 MediClin Robert Janker Klinik, Bonn, Germany; 13 Landeskrankenhaus, Universitätsklinikum der Paracelsus Medizinischen Privatuniversität Salzburg, Salzburg, Austria; 14 Department of Radiation Oncology, Universitätsklinikum Freiburg, Freiburg, Germany; 15 partner site Tübingen, and German Cancer Research Center (DKFZ), German Cancer Consortium (DKTK), Heidelberg, Germany; 16 Department of Radiation Oncology, Universitätsklinikum Ulm, Ulm, Germany

**Keywords:** Prostate cancer, Low metastatic burden, Radiotherapy of the primary, Hormone sensitive, Palliative standard of care (SOC)

## Abstract

**Purpose:**

The DEGRO Expert Commission on Prostate Cancer has revised the indication for radiation therapy of the primary prostate tumor in patients with synchronous distant metastases with low metastatic burden.

**Methods:**

The current literature in the PubMed database was reviewed regarding randomized evidence on radiotherapy of the primary prostate tumor with synchronous low metastatic burden.

**Results:**

In total, two randomized trials were identified. The larger study, the STAMPEDE trial, demonstrated an absolute survival benefit of 8% after 3 years for patients with low metastatic burden treated with standard of care (SOC) and additional radiotherapy (RT) (EQD2 ≤ 72 Gy) of the primary tumor. Differences in the smaller Horrad trial were not statistically significant, although risk reduction in the subgroup (< 5 bone metastases) was equal to STAMPEDE. The STOPCAP meta-analysis of both trials demonstrated the benefit of local radiotherapy for up to 4 bone lesions and an additional subanalysis of STAMPEDE also substantiated this finding in cases with M1a-only metastases.

**Conclusion:**

Therefore, due to the survival benefit after 3 years, current practice is changing. New palliative SOC is radiotherapy of the primary tumor in synchronously metastasized prostate cancer with low metastatic burden (defined as ≤ 4 bone metastases, with or without distant nodes) or in case of distant nodes only detected by conventional imaging.

## Introduction

Prostate-targeted local treatments in synchronously metastasized prostate cancer include external beam radiotherapy (RT), radical prostatectomy with or without extended lymphadenectomy, transurethral resection, or brachytherapy, mostly in addition to androgen deprivation therapy (ADT). Most available data regarding oncological benefit from “debulking” of the primary come from retrospective analyses and were either conducted in well-selected patients or in patients with local symptoms, leading to heterogeneous results [1].

Stage IV prostate cancer comprises different prognostic groups (cN1, cM1a–c) [2], from oligometastatic nodal stages to different metastatic burdens. The term oligometastasis was described by Helman et al., who defined it as an intermediate state between local and systemic disease with limited tumor burden [3]. Currently, there is no interdisciplinary consensus available regarding the definition of oligometastasis in prostate cancer [4]. A general consensus on oligometastasis of ESTRO and ASTRO stated a threshold of 1–5 metastases. However, for prostate cancer it has not been defined whether pelvic nodes, paraaortic nodes, or bone metastases should be counted [5]. Trials on systemic treatment use definitions like CHAARTED or LATITUDE to differentiate between low and high metastatic burden [6, 7], Table 1. Low metastatic burden was defined in both definitions by the number/amount of bone metastases (M1b) and absence of visceral metastases (M1c), irrespective of the number of nodal (N1) or paraaortic nodes (M1a). Therefore, low burden is not an oligometastatic stage, but with 1–5 metastases (N1–M1b) there could be an overlap of both definitions.

**Table 1:** CHAARTED and LATITUDE criteria

Definition	Metastatic burden	Parameter
CHAARTED	Low	*No poor risk criteria*
CHAARTED	High	*≥* *4 bone metastases (≥* *1 beyond vertebral column and pelvis)* AND/OR *Visceral metastasis (M1c)*
LATITUDE	Low	*Maximal 1 risk criteria*
LATITUDE	High	*≥* *2 of the following criteria:* Gleason score ≥ 8 ≥ 3 bone metastases Visceral metastasis (M1c)

The SOC (standard of care) for stage IV comprises androgen deprivation therapy (ADT). Depending on the individual risk of progression, ADT is combined with either abiraterone, enzalutamide, apalutamide, or docetaxel according to current guidelines [8–11]. The STAMPEDE trialists reported in October 2018 a survival benefit of 8% after 3 years for patients with low metastatic burden receiving SOC and additional percutaneous irradiation (RT) of the primary only. Positive nodes or bone metastases were not irradiated [12]. The results of this large randomized trial led to a change of international guidelines and current practice [8, 13]. For this reason, the DEGRO expert commission on prostate cancer reviewed randomized trials and related literature to derive conclusions and open questions regarding this issue.

## Materials and methods

A literature review using the PubMed database was performed in April 2021. The search strategy included the terms “metastatic prostate cancer” AND “radiotherapy.” In total, 4164 results were shown and filtering for “randomized controlled trials” reduced the number to 267 articles with 96 published in the last 5 years. The second search strategy comprised the terms “prostate cancer” AND “oligometastases” AND “radiotherapy,” leading to 122 results with 5 randomized controlled trials all published in the last 5 years.

Original articles of randomized trials in English were included. Inclusion criteria were primary prostate cancer, synchronous metastatic disease with limited burden, and radiotherapy of the primary with a palliative radiation dose. ACM, DZ, and TW selected the publications for inclusion. The Prostate Cancer Expert Panel of the German Society of Radiation Oncology (DEGRO) and the Clinical Trialist’s Working Party for Radiation Oncology in affiliation with the German Cancer Society (DKG-ARO) discussed the results in April and October 2021 and developed treatment recommendations.

## Results

### Literature search

The literature search for treatment of synchronous metastatic prostate cancer with treatment of the primary yielded two randomized controlled trials, the STAMPEDE trial [12] and the much smaller randomized Horrad trial by Boeve et al. [14]. Therefore, we decided to primarily present the original results of the STAMPEDE trial followed by the Horrad trial and related papers [12, 14–16]. Additional local treatment of positive lymph nodes or metastasis-directed RT was not performed and is currently under investigation.

### Patient characteristics and staging

#### STAMPEDE trial

From 2013 to 2016, men (*n* = 2061) with newly diagnosed metastatic prostate cancer confirmed by bone scan and soft tissue imaging (mainly CT abdomen/pelvis) were included. A PET scan was not performed. Imaging should have been done within 12 weeks of starting ADT (lifelong GnRH-agonists or orchiectomy). Patients had received no previous radical treatment, presented without significant cardiovascular disease, and had no contraindications to radiotherapy. Metastatic burden was classified according to the CHAARTED-definition [6]. High metastatic burden was defined as 4+ bone metastases with one or more outside the vertebral bodies or pelvis, or visceral metastases, or both. All other patients were considered to have low metastatic burden [12]. Patients (median age 68 years) were represented with 90% T3+ cancers, 64% N1 disease, and 29% distant nodes (M1a). Bone metastases were detected in 89%, visceral metastases in 10%. Initial median PSA level before ADT was ~97 ng/mL (RT arm: 1‑11156). A Gleason score of 8–10 was detected in 79% (*n* = 1630/2061). WHO performance score was 0 in 71% (*n* = 1466). Low metastatic burden was classified in 42% of patients (*n* = 819/1939, 122 patients unclassified).

#### HORRAD trial

From 2004–2014, men (*n* = 432) with PSA > 20 ng/ml (median: 142 ng/ml) with bone metastases on bone scan were included. In contrast to the STAMPEDE trial, patients were subdivided into < 5, 5–15, or more bone lesions.

### Treatment and randomization

#### STAMPEDE trial

Docetaxel had been given in 18% of patients since 2015 in addition to lifelong ADT. RT was randomized 1:1 to SOC. Two treatment schedules were allowed: 55 Gy in 20 fractions (52%) and 36 Gy in 6 weekly fractions (48%). Planning target volume included the prostate with a 10-mm (posteriorly 8 mm) margin. RT was started as soon as practicable; for patients with docetaxel treatment, RT was started 3–4 weeks after the last cycle. Metastatic sites received no RT.

#### HORRAD trial

RT schedules (comparable PTV definition) of either 70 Gy in 35 daily fractions or 57.76 Gy in 19 fractions (three times per week) were used. SOC consisted of LHRH (initially 4 weeks with bicalutamide 50 mg as flare-up reduction) until death. Metastatic sites received no RT. Palliative radiation schedules are summarized in Table 2.

**Table 2:** Radiation treatment schedules and EQD2 according to initial estimates of Brenner and Hall [23] as well as recent calculations of a meta-analysis by Vogelius and Bentzen [24]

Treatment schedule	EQD2, Gy (α/β = 1.5) [1]	EQD2, Gy (α/β = 2.7) [2]
36 Gy in 6 fractions (weekly)	77.14	66.64
55 Gy in 20 fractions	66.79	63.78
57.76 Gy in 19 fractions (thrice weekly)	74.92	70.54
70 Gy in 35 fractions	70	70

*EQD2* equivalent dose in 2‑Gy fractions

### Toxicity

#### STAMPEDE trial

High-grade late bladder and bowel toxicity (RTOG G3+) was low in both arms, with 1% vs. 4%, respectively. CTCAE toxicity (any abnormality) of grade 3 or worse was similar in both arms and dominated by ADT-related side effects. Time to first grade 3 event did not differ between SOC or SOC + RT (*p* = 0.97). After 2 years, 15% of the control group and 13% of the experimental group reported at least a grade 3 event. Considering different RT schedules, there was no significant difference between the approaches in terms of higher-grade acute toxicity (insignificantly more acute RTOG G3+ bowel toxicity for the 4‑week schedule). However, the incidence of acute RTOG toxicity (all grades) was higher for the daily schedule (bladder: 71% vs. 65% and bowel: 62% vs. 47%). Subanalysis for the late toxicity of different RT schedules was not reported.

#### HORRAD trial

Toxicity was not reported.

### Response to treatment

#### STAMPEDE trial

Reported clinical endpoints were calculated from the date of randomization. Primary endpoint was overall survival (OS), secondary endpoints included progression-free survival (PFS, without biochemical events) and metastatic PFS (defined as time to new metastases or progression of existing metastases or death). Biochemical failure (BF) was defined as rise above 50% (at least 4 ng/mL) of the lowest PSA level reported within 24 weeks after enrolment. In case of no fall of 50%, BF was defined at time zero.

#### STAMPEDE trial unselected patients

With a median follow-up of 37 months, SOC vs. SOC + RT resulted in unselected patients (low and high metastatic burden) in no survival benefit at 3 years (3-year OS: 62%, *p* = 0.451). Failure-free survival was significantly improved in unselected patients with RT (HR 0.76, 95% CI 0.68–0.84, *p* < 0.0001). The impact of the 4‑week schedule (HR 0.69, 95% CI 0.59–0.80, *p* < 0.0001) seemed to be higher than that of the weekly schedule (HR 0.85, 95% CI 0.73–0.99, *p* = 0.033) compared to controls. There was an insignificant trend favoring the 4‑week schedule (*p* = 0.27) regarding overall survival compared to controls (HR 0.86 vs. 1.01). Due to insufficient evidence, an OS effect was not reported for the weekly schedule.

#### STAMPEDE trial selected patients

In patients with low metastatic burden only, a prespecified subgroup analysis demonstrated a survival benefit at 3 years (81% vs. 73%, HR 0.76, 95% CI: 0.68–0.84), improved FFS (50% vs. 33%, HR 0.59, 95% CI: 0.49–0.72), improved PFS (63% vs. 58%, HR 0.78, 95% CI: 0.63–0.98), and improved PCSS (86% vs. 79%). Symptomatic local event-free survival was comparable (72% vs. 65%, HR 0.82, 95% CI: 0.64–1.05) in both groups.

#### HORRAD trial

Time to PSA progression was improved with additional RT (15 vs. 12 months; logrank *p* = 0.02 but adjusted HR nonsignificant). OS did not differ between the two arms (45 vs. 43 months). The HR for patients with < 5 bone metastases receiving RT was equivalent to STAMPEDE 0.68 (95% CI 0.42–1.10) but nonsignificant due to sample size.

## Discussion

The STAMPEDE trial and the meta-analysis of both trials demonstrated no benefit for unselected metastatic prostate cancer patients. However, in a predefined large subgroup analysis, a benefit for OS and biochemical control was shown for patients with low metastatic burden according to the CHAARTED criteria. Therefore, RT of the primary for low metastatic burden represents a palliative cytoreductive treatment (any T, any N, distant nodes allowed [M1a]) ± limited bone metastases (M1b); i.e. nodes and metastases are treated by systemic therapy. Local RT led to improved survival and is now SOC in this clinical stage. The pooled analysis of both studies substantiated this finding [16]. A recent exploratory analysis of the STAMPEDE trial by Ali et al. demonstrated that patients with non-regional nodes alone as well as patients with up to three bone metastases had the greatest benefit of local RT in addition to previous SOC [15]. In general, a low burden of 3–4 (nonirradiated) bone metastases (*n* = 3 CHAARTED/STAMPEDE, *n* = 4 HORRAD/STOPCAP meta-analysis [16]) identified the subgroup benefiting from prostate RT. Therefore, it is reasonable to investigate whether this effect might be boosted by additional treatment of all lesions. For the situation of M1a-only metastases, local RT of the primary can be recommended with a lower level of evidence (finding of the subgroup analysis of Ali et al.) [15].

### Which classification and staging should be used?

Currently, we only have evidence for the fact that addition of RT to the primary in synchronous limited metastatic prostate cancer improves survival when the CHAARTED criteria are applied. It is essential to distinguish this patient group from PET-CT-staged patients with oligometastasis, i.e., a limited number of nodal and/or bony metastases. For postoperative treatment of patients with oligo-nodes, pelvic RT became an option in current guidelines. The described “low metastatic burden” patient population defined by conventional staging (bone scan, CT) included clearly more patients with multiple (i.e., nodal or paraaortic) metastases (no limit of nodal metastases) compared to patients with PET-CT-staged oligometastatic disease. In addition, in patients with low metastatic burden, pelvic metastases were treated by systemic therapy only and not by radiotherapy. Therefore, the two trials cannot unequivocally serve as a guideline in the PET era for oligometastatic disease, where local treatment of all lesions is under investigation.

On the other hand, we agree with Parker et al. to not exclude prostate cancer patients from palliative local RT, if PET staging detected more metastases than comprised by low metastatic burden on conventional staging [12].

The current update of the German S3 guideline defines oligometastasis as having < 5 bone metastases and no visceral metastases detected by conventional staging, which is based on the pooled results of both trials according to the STOPCAP meta-analysis demonstrating a 3-year overall survival benefit of 7% in this subgroup [16]. However, this finding is based on a secondary analysis of a phase III trial and does not demonstrate the primary or secondary endpoint.

### Do we have prognostic subgroups?

The analysis of prognostic factors of metastasized patients led to the CHAARTED criteria and to the LATITUDE criteria to trigger systemic treatment, Table 1; [7, 12]. The analysis of combined risk factors in a large cohort suggested that patients with both paraaortic nodes and bony metastases have the same prognosis as patients with visceral metastases (M1a + M1b = M1c). However, this was not clinically tested and significant confounders (staging method) might be present [2]. Currently, there is no consensus on further prognostic subgroups for metastatic prostate cancer beyond the CHARTED or LATITUDE definition [17–19].

### Which radiotherapy schedule should be used to treat the primary?

In STAMPEDE, 55 Gy in 20 fractions or 36 Gy in 6 weekly fractions were used. The HORRAD trial employed either 70 Gy in 35 fractions or 57.76 Gy in 19 three weekly fractions. The authors state that this schedule represents an older radiotherapy standard. Therefore, Parker et al. recommend applying the current standard of 60 Gy in 20 fractions. However, this CHiP standard and other randomized trials were not tested in locally advanced disease or after transurethral resection, and there remains a risk of slightly increased GU toxicity with hypofractionation. The current German S3 guideline [9] recommends local treatment with moderately hypofractionated or conventionally fractionated RT of the primary with an EQD2 of up to 72 Gy, in line with EQD2 (70–74 Gy) of the HORRAD trial (Table 2; [14]).

We discussed this point and recommend a practical approach depending on pre-existing comorbidities, previous surgery (such as TUR-P), and individual life expectancy. The moderately hypofractionated regimen (57–57.76 Gy in 19 fractions [F]) might be suitable in patients without TUR‑P and with IPSS ≤ 12 (achieved after neoadjuvant ADT) [20]. This would acknowledge the experience with patients of the CHiP trial with slightly increased genitourinary toxicity [21]. Normofractionated schedules to 70–72 Gy (35–36 F) or hypofractionated schedules with lower EQD2 (36 Gy in 6 weekly F or 55 Gy in 20 F) might be more suitable in patients with an increased risk of toxicity (after TUR‑P, urinary obstruction), since evidence of low toxicity can be derived from both trials for locally advanced disease.

Overall survival data of the PEACE-1-trial comparing triplet therapy SOC (ADT ± docetaxel) ± abiraterone ± RT with 74 Gy [37F] are not yet mature. They should further substantiate the role of each component, including a schedule with the highest radiation dose.

### Additional local treatment of distant metastases?

Additional local treatment of metastases is currently under investigation and optimal patient selection, i.e., maximal number of distant lesions (nodes ± bone metastases) needs to be defined. This is not addressed by the two discussed trials. Both trials contain patients with low metastatic burden but not oligometastatic disease, where metastases-directed treatment might be reasonable to improve progression-free survival [22] and should be discussed individually in an interdisciplinary tumor board.

## Conclusion

Radiotherapy of the primary tumor is a new standard for metastatic prostate cancer with low metastatic burden according to the CHAARTED criteria. The update of the German S3 guideline summarizes criteria for low burden according to published trials and the STOPCAP meta-analysis as up to 4 bone metastases (no visceral metastases). In these patients, palliative prostate RT should be performed up to 72 Gy (EQD2) using established hypofractionated or normofractionated schedules considering individual patient characteristics (i.e., obstruction, prostate volume, OARs etc.) [23, 24].

The new palliative standard of care (SOC) is radiotherapy to the primary tumor in synchronously metastasized prostate cancer with low metastatic burden defined as ≤ 4 bone metastases (with or without distant nodes) or—with a lower level of evidence—in case of distant nodes only.
